# A lensed fiber Bragg grating-based membrane-in-the-middle optomechanical cavity

**DOI:** 10.1038/s41598-022-08960-0

**Published:** 2022-03-23

**Authors:** Joris Baraillon, Boris Taurel, Pierre Labeye, Laurent Duraffourg

**Affiliations:** Commissariat à l’Energie Atomique, LETI, Université Grenoble Alpes, 38054 Grenoble, France

**Keywords:** Optomechanics, Fibre optics and optical communications, Optical sensors

## Abstract

Optomechanical systems benefit from the coupling between an optical field and mechanical vibrations. Fiber-based devices are well suited to easily exploit this interaction. We report an alternative approach of a silicon nitride membrane-in-the-middle of a high quality factor ($10^6$–$10^7$) Fabry–Perot, formed by a grating inscribed within a fiber core as an input mirror in front of a dielectric back mirror. The Pound–Drever–Hall technique used to stabilize the laser frequency on the optical resonance frequency allows us to reduce the low frequency noise down to ${4}\,{{\mathrm{kHz}}/\sqrt{\mathrm{Hz}}}$. We present a detailed methodology for the characterization of the optical and optomechanical properties of this stabilized system, using various membrane geometries, with corresponding resonance frequencies in the range of several hundred of ${\mathrm{kHz}}$. The excellent long-term stability is illustrated by continuous measurements of the thermomechanical noise spectrum over several days, with the laser source maintained at optical resonance. This major result makes this system an ideal candidate for optomechanical sensing.

## Introduction

Cavity optomechanics explores the mutual interaction of electromagnetic radiation and mechanical displacement using optical and mechanical resonators. This coupling have been explored in a wide variety of bulk systems, from the Fabry–Perot with a suspended micromirror^1^ to the mechanical membrane-in-the-middle (described as MIM hereafter) of such cavity^2^. Multiple integrated nanoscale devices have also been implemented, such as suspended microdisks^3^, photonic and phoxonic crystal systems^4–6^, or whispering gallery mode resonators-based setups^7–11^.

The past two decades have also seen the appearance of numerous fibered optomechanical systems for various applications. Using highly reflective Bragg grating coated concave fiber end facets formed by $\hbox {CO}_2$ laser machining (laser ablation of the fiber ends)^12^, high to ultra-high finesse (between $10^3$ and $10^5$) fiber-based optomechanical cavities can be constructed. These setups are excellent basis for the “mechanical resonator in the middle” system, where the mechanical resonator properties and the optical finesse of the cavity can be optimized independently (unlike the Fabry–Perot with a suspended mirror). Research has been conducted on carbon nanorod^13^ and nanotube^14^ in the middle of such cavities, with optical measurement of their Brownian motion. Other groups have worked on the introduction of a high quality factor silicon nitride membrane: the first study on this fiber-based MIM setup performed observation of dynamical backaction with dispersive optomechanical interaction (shift of the optical resonance frequency induced by the mechanical displacement), with optically induced mechanical resonance frequency shift (optical spring effect) and optomechanical damping^15^. Similar configurations have been constructed ever since, with the associated dynamical backaction observations, with various other interesting properties, from the optically mediated mechanical mode hybridization^16^, to the additional dissipative optomechanical interaction (shift of the optical losses with the mechanical displacement)^17,18^. Finally, the group of Eyal Buks has worked on various Fiber Bragg Grating (FBG) based optomechanical cavities. Their systems are composed of a highly reflective Bragg grating inscribed on the core of a fiber, which serves as a static input mirror of a Fabry–Perot cavity at $\lambda ={1.55}\,{\upmu \text {m}}$, in front of a suspended metallic back mirror (rectangular or beam structure). The optical finesse in this situation is relatively low (of the order of 10), due to a lower FBG reflectivity, in comparison to the ultralow loss Bragg coated fiber tips. They designed various configurations, with the metallic beam directly fixed on the polished fiber end, inducing a passive alignment^19–21^, or with the light focused on the micromechanical resonator by means of a graded index lens spliced to the end facet of the fiber^22,23^. Bolometric self-sustained oscillations above a certain input threshold have been observed, using the heating of the metallic mirror due to optical absorption. To the best of our knowledge, these are the only studies on FBG-based optomechanical cavities.

In this article, a novel FBG-based optomechanical system is presented. An easy-to-build external lensed FBG-based MIM setup using a silicon nitride membrane, a piezo-electric material and a dielectric back mirror is proposed. A detailed and extensive study of this low-finesse optomechanical cavity is performed. Basic optical characterizations allow a rough estimation of the dispersive and dissipative interaction. Using a feedback loop to maintain the laser source at optical resonance, a complete noise analysis of these frequency stabilized cavities is done, with an optimization of the correction parameters. An empirical method specific to this system is set up to easily and rapidly identify the vibration mode shapes, allowing measurement of multiple thermomechanical responses of membranes with various geometries. Miscellaneous optomechanical and thermal properties are finally investigated: in particular, the long term stability of the system is illustrated by continuous measurements of the thermomechanical motion with the laser maintained at optical resonance. This excellent stability makes our MIM setup an ideal candidate for optomechanical sensing.

**Figure 1:**
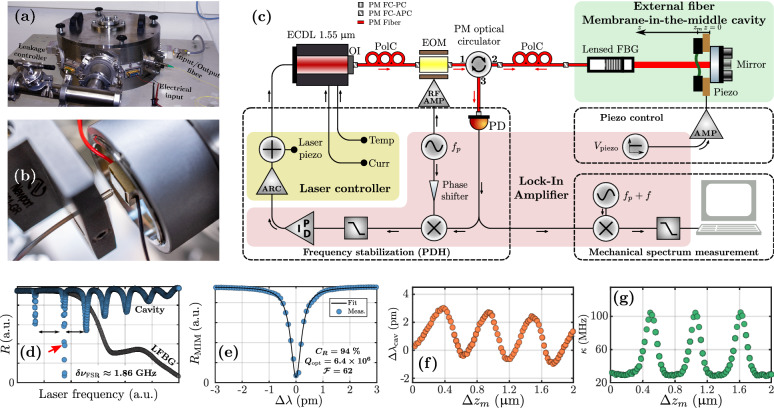
The lensed FBG-based optomechanical MIM cavity. (**a**) Picture of the vacuum chamber. (**b**) Picture of the cavity itself with the silicon nitride membrane passively aligned with the dielectric mirror using a piezo-electric element as a spacer in between. (**c**) Schematic of the experimental setup used for the thermomechanical characterization with a PDH frequency stabilization scheme. ARC: Analog Remote Control, AMP: Amplifier, ECDL: External-Cavity Diode Laser, EOM: electro-optic modulator, FBG: Fiber Bragg Grating, FC-PC/FC-APC: Fiber Coupled connectors with Physical Contact/Angled Physical Contact, OI: Optical Isolator, PD: PhotoDetector, PID: Proportionnal-Integral-Derivative, PolC: Polarization Controller, PM: Polarization Maintaining. (**d**) Typical optical spectra in reflection of the cavity and comparison with the optical response of the lensed FBG (LFBG) alone. The corresponding free spectral range $\Delta \nu _{\text {FSR}}$ is indicated with double arrows. (**e**) Resonance peak of highest contrast [indicated by the red arrow in (**d**)] fitted by a Lorentzian shape function (simplified Airy function, suitable because of the high optical quality factor) to extract to the contrast $C_R$, the quality factor $Q_{\text {opt}}$ and the optical finesse ${\mathcal {F}}$. (**f**), (**g**) Measured resonance wavelength shift $\Delta \lambda _{\text {cav}}$ (orange) and decay rate $\kappa$ (green) as a function of the membrane relative position along the cavity axis. The initial position ($\Delta z_m = 0$) is the situation without any voltage applied on the piezo-electric material (minimum position $z_1 = {3.25}\,{\mathrm{mm}}+L_m/2$ where $L_m$ is the membrane thickness).

## Results

### Basic optical characterizations

The optomechanical setup of interest is an external lensed FBG-based MIM cavity. The reader can refer to Methods for more detail on the optical elements, the cavity construction and the alignment setup. The uniform FBG^24^ serves as a highly reflective input mirror, at the working wavelength of $\lambda ={1.55}\,{\upmu \text {m}}$, with a spectral bandwidth of ${0.5}\,{\mathrm{nm}}$. The back mirror of the cavity is a highly reflective plane broadband dielectic mirror. In order to collimate light out of the fiber, and to drastically reduce the coupling losses between the beam reflected by this mirror and the fiber guided mode, a gradient-index (GRIN) lens was bound directly on the fiber tip. It enables a coupling efficiency around ${90}\,{\%}$ for a typical distance between the lens and the mirror at ${5}\,{\mathrm{mm}}$. The membrane is passively aligned with the back mirror using a piezo-electric material as a spacer in between. Squared silicon nitride membranes from Norcada (low stress SiN or stoichiometric high stress $\hbox {Si}_3\hbox {N}_4$, optical index at $n=1.9963$ at $\lambda ={1.55}\,{\upmu \text {m}}$)^25^ with a thickness $L_m=30$ or ${50}\,{\mathrm{nm}}$, and a lateral dimension $a=0.5$ or ${1}\,{\mathrm{mm}}$, are used. The difference between the devices presented hereafter only comes from the membrane geometry. The cavity length $L_{\text {cav}}\approx {13}\,{\mathrm{mm}}$, mostly fixed by the lensed FBG itself, remains indeed similar from one device to another. The main usage of the piezoelectric component is to precisely move the membrane along the cavity axis, with a sensitivity around ${22.4}\,{\mathrm{nm/V}}$ *i.e.* $1.5\times 10^{-2}\,\lambda {/{\mathrm{V}}}$. The whole cavity is aligned with a homemade setup, and placed in a vacuum chamber to minimize air damping (see Fig. 1a, b). The fiber-based cavities are characterized optically in reflection using the bench sketched in Fig. 1c. A typical reflection response ($\hbox {Si}_3\hbox {N}_4$, $L_m={50}\,{\mathrm{nm}}$, $a={1}\,{\mathrm{mm}}$), compared to the FBG spectrum, is displayed in Fig. 1d. The free spectral range $\delta \nu _{\text {FSR}}$ is quantified between 1.5 and ${2.5}\,{\mathrm{GHz}}$ for all our devices. The chosen optical resonance frequency $f_{\text {cav}}$ maximises the optical contrast: the peak of interest (see Fig. 1e) is fitted using a Lorentzian function, to quantify the contrast $C_R$, the quality factor $Q_{\text {opt}}$, and finally the finesse ${\mathcal {F}}$. Each quantity varies for all our device in these respective ranges: $C_R\in [80, 98]\,{\%}$, $Q_{\text {opt}}\in [10^6, 10^7]$, and ${\mathcal {F}}\in [50, 150]$. By moving the membrane away from the mirror with the piezo while measuring the spectral response, the periodic evolution of the resonance condition $\Delta \lambda$ and the cavity bandwidth (or decay rate) $\kappa$ with the membrane position along the cavity axis (due to an imbalance between the optical resonances of the two sub-cavities which hybridize through the membrane transmission) is verified^2^ (see Fig. 1f, g). The period is close to ${0.75}\,{\upmu \text {m}}$ i.e. a half-wavelength range which corresponds to the gap between nodes and antinodes of the stationnary intra-cavity field. The amplitude of variation is always around $3-{4}\,{\mathrm{pm}}$ in wavelength for the resonance condition and varies around ${100}\,{\mathrm{MHz}}$ for the decay rate. The variation of losses observed on the cavity bandwith curve can be attributed to the alignment and fiber coupling configurations that are not completly preserved when moving the membrane along the cavity axis. Note that our setup is actually very similar to a membrane-at-the-edge (MATE) system^26,27^, considering the lens assembly length above ${7}\,{\mathrm{mm}}$. This geometrical property results in a slight asymmetry in the variation of resonance condition (due to a stronger imbalance between the resonances of the two sub-cavities). The cavity lenght in the ${\mathrm{cm}}$ range is quite large compared to other fiber-based optomechanical cavities from the literature^13–18^, which enhances this effect (maximum variation of the resonance condition inversely proportional to the cavity length^27^). These measurements indicate potential dispersive and dissipative interaction (both estimated with the maximum slope in the ${\mathrm{MHz/nm}}$ range), and help to identify ideal membrane positions to optimize the dynamical couplings *i.e.* the optical sensitivity to mechanical vibrations.

**Figure 2:**
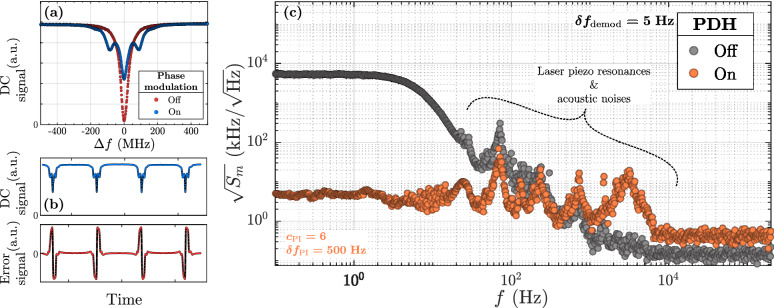
Noise analysis of the frequency stabilized fiber-based optomechanical cavity. (**a**) Typical spectral response (or DC signal) in reflection as a function of the frequency detuning $\Delta f$. For this example, the cavity bandwidth is around $\kappa = {42.1}\,{\mathrm{MHz}}$, the modulation frequency is $f_p = {75}\,{\mathrm{MHz}}$, and the phase modulation depth is close to the optimal value of $\beta \approx 1.08$. (**b**) Time acquisition of the DC and the corresponding error signal, with a forward/backward continuous laser wavelength scan (for qualitative purpose). (**c**) Optimized low frequency noise spectrum (or power spectral density) of the reflected signal after the noise analysis. The unit is in ${{\mathrm{kHz}}/\sqrt{{\mathrm{Hz}}}}$ which corresponds to shift of the optical resonance frequency normalized by the demodulation bandwidth $\delta f_{\text {demod}}$. The proportionnal factor $c_{\text {PI}}$ and the correction bandwidth $\delta f_{\text {PI}}$ are indicated. The noise spectrum is displayed with and without the PDH closed loop for comparison purpose.

### Frequency stabilization and noise analysis

The Pound–Drever–Hall (PDH) method^28^, widely used, especially in the optomechanical research community^2,29–38^, is implemented to stabilize the laser source on the resonance of the optical cavity. Using an electro-optic modulator (EOM) for the phase modulation, a photoreceiver in reflection, a lock-in amplifier (LIA) for the modulation/demodulation process, a signal proportionnal to the derivative of the reflection spectrum is generated (see Fig. 2a, b). The linear part can therefore be used as an error signal to dynamically adjust the laser emission frequency with a Proportionnal-Integral-Derivative (PID) corrector. The closed loop stabilizes the laser frequency on the optical resonance frequency, and corrects any frequency fluctuations until a limit, mainly fixed by the chosen integration and PID bandwidths. The experimental setup is illustrated in Fig. 1c (see “Methods” for more details on the implementation of the PDH closed loop). In practice, only a PI corrector is used (without the derivative term), with the following features:

1 $$\begin{aligned} P_{\text {PDH}}&= \text {sgn}(\varphi ) c_{\text {PI}}/s_{\text {err}}, \end{aligned}$$

2 $$\begin{aligned} I_{\text {PDH}}&= 2\pi \delta f_{\text {PI}} P_{\text {PDH}}, \end{aligned}$$

where $\text {sgn}$ is the sign function, $\varphi$ is the phase shift between the LIA oscillator and the reflected signal measured by the photodetector, $c_{\text {PI}}$ is a proportionnal factor used for an optimization process, $s_{\text {err}}$ is the error signal slope in the linear part, and $\delta f_{\text {PI}}$ is the correction bandwidth. Before any stabilization, the ideal modulation parameters are found. The evolution of the error signal slope as a function of the modulation depth $\beta$ and frequency $f_p$ is analyzed. Firstly, there is an optimal depth for which the error sensitivity is maximized ($\beta \approx 1.08$). Secondly, $f_p$ is chosen in the high frequency regime *i.e.* above the cavity bandwidth $\kappa$^28^. Typical parameters for the previously presented cavity are $\kappa \approx {42.1}\,{\mathrm{MHz}}$ and $f_p={75}\,{\mathrm{MHz}}$. This preliminary analysis allows the system to be placed in an ideal situation to improve its sensitivity to frequency fluctuations induced on the optical resonance. The error sensitivity $s_{\text {err}}^{{\mathrm{W/Hz}}}$ (measured output power variation per resonance frequency shift) is given by:

3 $$\begin{aligned} s_{\text {err}}^{{\mathrm{W /m}}} = \dfrac{c}{\lambda ^2} s_{\text {err}}^{{\mathrm{W /Hz}}} = \dfrac{c}{\lambda ^2}G_{\text {PD}}\,{\mathcal {R}}_{\text {PD}} s_{\text {err}}^{{\mathrm{V /Hz}}} \end{aligned}$$

where *c* is the light velocity, $G_{\text {PD}}$ is the photodetector gain ($10^2\,{\mathrm{V/A}}$), ${\mathcal {R}}_{\text {PD}}$ is its responsivity (${0.98}\,{\mathrm{A/W}}$) and $s_{\text {err}}^{{\mathrm{V/Hz}}}$ is the error slope. One have, for this specific example, $s_{\text {err}}^{{\mathrm{W/m}}}={29.3}\,{\mathrm{mW}/{\mathrm{pm}}}$. This is typical, and stays in the same order of magnitude from one cavity to another, as it mainly depends on the cavity bandwidth, similar between all the devices.

After setting on the closed loop, a noise analysis is performed with an optimization procedure: $c_{\text {PI}}$ is swept (between 0.8 and 8–9), as wel as $\delta f_{\text {PI}}$ (between 100 and ${1}\,{\mathrm{kHz}}$), while measuring the noise power spectral density (PSD) of the reflected signal, denoted by $S_m$. Note that the PI bandwidth cannot be chosen above ${1}\,{\mathrm{kHz}}$ because of a resonance of a piezo electric element in the laser source in the ${\mathrm{kHz}}$ range (see “Methods”). The PSD is recovered by demodulating the signal measured by the photodetector with a second oscillator of the LIA at $f_p + f$, where *f* varies between ${0.1}\,{\mathrm{Hz}}$ and ${200}\,{\mathrm{kHz}}$. As an example, an optimized noise PSD of one of the fiber optomechanical cavities (with a $\hbox {Si}_3\hbox {N}_4$ membrane) is displayed in Fig. 2c (“PDH on”). The measured PSD in ${\hbox {V}^2/{\mathrm{Hz}}}$ is converted into a frequency PSD in ${\hbox {Hz}^2/{\mathrm{Hz}}}$ (cavity resonance frequency shift normalized by the integration bandwidth) using the error signal sensitivity. Considering this spectrum as pure noise -which is consistent with our application where the measurable quantity of interest, *i.e.* the mechanical signal of the membrane, oscillates at higher frequencies- three frequency regimes are identified. First, the low frequency regime (below ${50}\,{\mathrm{Hz}}$) is associated with the efficiency of the lock: it quantifies the frequency noise of the system, from laser and cavity fast drifts. The intermediate regime (between 50 and ${100}\,{\mathrm{kHz}}$) is characterized by acoustic noises and resonances of the laser piezo, which could be reduced by using an efficient vibration-damping optical table. Finally, the high frequency regime ($>{100}\,{\mathrm{kHz}}$) corresponds to the noise floor for the measurement of the optical resonance frequency fluctuations (oscillating above ${100}\,{\mathrm{kHz}}$) induced by any sources. The typical optimal noise spectrum (“PDH on”) is compared to the spectrum without feeding the laser piezo with the correction signal (“PDH off”) in Fig. 2c. The PDH technique is intended to stabilize the system over long term. One can indeed clearly see a decrease of the low frequency noise (below ${50}\,{\mathrm{Hz}}$) of more than 2 decades, which indicates a stabilization between the laser and the cavity frequency drifts. In addition, it has a negligible influence on the background noise in the high frequency regime (above ${100}\,{\mathrm{kHz}}$). The setup is then adapted for long term measurement and for future sensing applications, which would require long acquisition time and a long-term stability.

**Figure 3:**
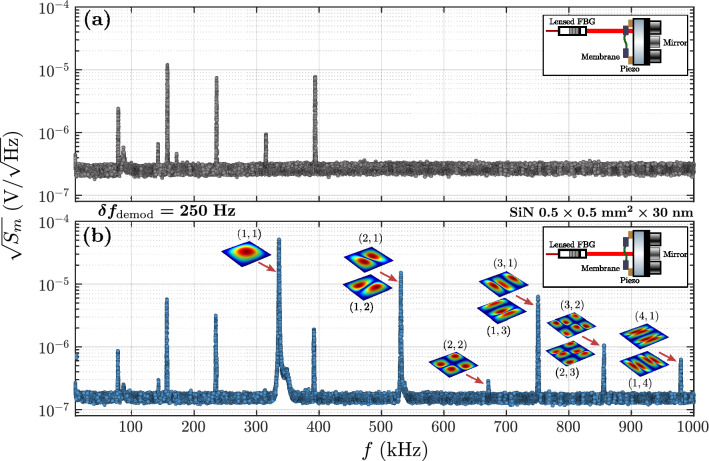
Identification of the resonant mechanical modes of a SiN membrane. (**a**) Measured wide range reference thermomechanical spectrum of the silicon frame (in gray) and (**b**) of the SiN membrane (square geometry $0.5\times {0.5}\,{\mathrm{mm}}\times {30}\,{\mathrm{nm}}$, in blue), in voltage unit (optical PSD). The mechanical modes of interest (i.e. of the membrane) are identified by comparison with the reference spectrum and shown with red arrows. The (*m*, *n*) modal indices are indicated. The shapes of each identified mode, calculated by finite element modeling simulation, are depicted. The insets show a schematic of the optical cavity for each measurement.

**Table 1:** Measured resonance frequencies in ${\mathrm{kHz}}$ (for two membranes) with large range mechanical sweep below ${1}\,{\mathrm{MHz}}$, and deduced intrinsic in-plane tension.

	$${\mathcal {T}}{({\mathrm{MPa}} )}$$	(m,n)					
		(1,1)	(2,1)	(2,2)	(3,1)	(3,2)	(4,1)
			(1,2)		(1,3)	(2,3)	(1,4)
Theory	$2\rho _V\,a^2f_{11}^2$	$f_{11}$	$1.581f_{11}$	$2f_{11}$	$2.236f_{11}$	$2.55f_{11}$	$2.915f_{11}$
(Modal analysis)							
SiN	177.9	335.9	531.2	671.5	750.8	856.5	979.5
(Fig. 3)			($1.581f_{11}$)	($1.999f_{11}$)	($2.235f_{11}$)	($2.550f_{11}$)	($2.916f_{11}$)
Si$_3$N$_4$	1002.3	397.6	628.6	795.4	889.7	$\backslash$	$\backslash$
($1\times {1}\,{\mathrm{mm}^2}\times {50}\,{\mathrm{nm}}$)			($1.581f_{11}$)	($2.001f_{11}$)	($2.238f_{11}$)		

Theoretical values of gap with the fundamental mode are indicated^39^. $\rho _V$ is the mass density and *a* the width of the membrane.

### Thermomechanical characterization

The thermomechanical characterizations are conducted using the optimized frequency stabilized external lensed FBG-based MIM optomechanical cavities in vacuum environment (pressure between $10^{-6}$ and $10^{-5}\,{\mathrm{mbar}}$) and by solely exploiting the dispersive interaction (shift of the optical resonance frequency induced by the mechanical vibrations). Before any calibration, the membrane resonance peaks have to be identified within the parasitic silicon frame thermal resonances. An efficient and fast empirical method is therefore set up to isolate the reference silicon frame spectrum. The Fig. 3 illustrates the method for a ${30}\,{\mathrm{nm}}$ thick SiN membrane. It consists of the acquisition of two large frequency range spectra using the PDH stabilization on two different optical cavities: one with the lensed FBG aligned on the frame and the other on the membrane surfaces (see schematics on insets of each graph). For both spectra ((a) frame and (b) membrane itself), the demodulation bandwidth $\delta f_{\text {demod}}$ is chosen high enough (${200}\,{\mathrm{Hz}}$), to quickly perform the identification, and with a large amount of measurement points, to sufficiently resolve all resonance peaks. The comparison of the two spectra leads to a clear identification of the mechanical modes of the membrane. The identified frequencies $f_{m,n}$ for this low-stress ${30}\,{\mathrm{nm}}$ SiN and for a high-stress ${50}\,{\mathrm{nm}}$ thick $\hbox {Si}_3\hbox {N}_4$ membrane are summarized in Table 1. The corresponding in-plane tension ${\mathcal {T}}$ is retrieved using the usual theory^39^.

**Figure 4:**
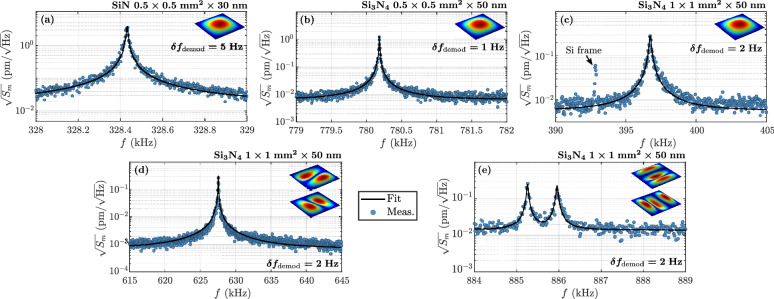
Observation of the membrane Brownian motion with a frequency stabilized lensed FBG-based MIM cavity. Calibrated fundamental thermomechanical spectrum of a (**a**) SiN ($0.5\times {0.5}\,{{\mathrm{mm}}^2}\times {30}\,{\mathrm{nm}}$), (**b**) $\hbox {Si}_3\hbox {N}_4$ ($0.5\times {0.5}\,{{\mathrm{mm}}^2}\times {50}\,{\mathrm{nm}}$), (**c**) $\hbox {Si}_3\hbox {N}_4$ ($1\times {1}\,{{\mathrm{mm}}^2}\times {50}\,{\mathrm{nm}}$) membrane. (**d**), (**e**) Thermomechanical spectrum of two higher order and degenerate modes. A removal of degeneracy is observed on the last spectrum.

The Brownian motion of multiple membranes with various geometries can then be studied. A thermomechanical calibration is performed to convert the measured optical PSD from voltage to displacement units, using the procedure explained in Methods and based on the article of Hauer et al.^39^. Thus, the measured spectra nearby the fundamental resonance frequency of:

- A SiN membrane ($0.5\times {0.5}\,{{\mathrm{mm}}^2}\times {30}\,{\mathrm{nm}}$) in Fig. 4a,
- A $$\hbox {Si}_3\hbox {N}_4$$ membrane ($$0.5\times {0.5}\,{{\mathrm{mm}}^2}\times {50}\,{\mathrm{nm}}$$) Fig. 4b,
- A $$\hbox {Si}_3\hbox {N}_4$$ membrane ($$1\times {1}\,{{\mathrm{mm}}^2}\times {50}\,{\mathrm{nm}}$$) Fig. 4c.

are displayed. Each spectrum is fitted (black curves) using the thermomechanical model (see Eq. (5) in “Methods”). The deduced mechanical parameters, as well as the dispersive optomechanical coupling are gathered in Table 2. The dispersive single-photon coupling strength $g_0$ is also estimated^40^. The three displayed spectra are characterized by a high signal to noise ratio (SNR) of more than two orders of magnitude. The mechanical quality factor values vary between $10^3$ and $10^5$ which is coherent with a previous characterization performed on the Norcada’s membranes^41^. The dispersive optomechanical couplings are in the ${\mathrm{MHz/nm}}$ range. The deduced vacuum coupling strenghts are relatively low compared to other fiber optomechanical cavities (in the order of $10^3\,{\mathrm{Hz}}$)^14–18^. This is explained by the low optical finesse (${\mathcal {F}}\approx 50-150$ in comparison to, in the best case scenario, $10^5$)^16,17^, but also mostly by the large dimensions of the system (cm range) and the high effective mass (in the ${\mathrm{ng}}$ range). Note that the noise floor value, taken into account in the model, is not given, and sometimes underestimated, due to the fitting frequency range that is greatly reduced around the resonance of interest. One could in practice have measured the mechanical PSDs on a bigger frequency range, but the acquisition time would have rapidly increased to keep constant the frequency resolution for each spectrum. Although the noise floor is not precisely known, it does not influence the measured resonance frequency, quality factor, as well as the conversion factor from voltage to displacement units.

**Table 2:** Deduced parameters on the thermochanical spectra of the three membranes.

Material	SiN	Si$_3$N$_4$	Si$_3$N$_4$			
Geometry	$0.5\times {0.5}\,{{\mathrm{mm}}^2}$	$0.5\times {0.5}\,{{\mathrm{mm}}^2}$	$1\times {1}\,{{\mathrm{mm}}^2}$			
Thickness	${30}\,{\mathrm{nm}}$	${50}\,{\mathrm{nm}}$	${50}\,{\mathrm{nm}}$			
(*m*, *n*)	(1, 1)	(1, 1)	(1, 1)	(2, 1)/(1, 2)	(3, 1)	(1, 3)
$f_{m}\,$ ${(\mathrm{kHz})}$	328.4	780.2	396.7	627.6	885.26	885.95
$Q_{m}$	$4.7\times 10^4$	$1.1\times 10^5$	$2.93\times 10^3$	$1.29\times 10^{4}$	$2.39\times 10^{4}$	$2.16\times 10^{4}$
$g_{\text {om}}\,$ ${(\mathrm{MHz/nm} )}$	2.57	6.44	9.49	46.18	3.85	3.88
$g_{0}\,$ ${({\mathrm{Hz}} )}$	13.4	16.8	17.4	67.2	4.7	4.8

Some higher order mechanical resonances are then investigated, on the last $\hbox {Si}_3\hbox {N}_4$ membrane. Two spectra of two degenerate resonance modes are displayed in Fig. 4d, e (respectively, (2, 1)/(1, 2) and (3, 1)/(1, 3)). A clear removal of degeneracy is observed in the second case. This removal of degeneracy is somehow controlled using the piezo material whose deformation is not perfectly symmetrical, which induces variations of the membrane constraints. The effective in-plane tension perceived by each mode may vary, which differently modifies each resonance frequency. Note that this effect has also been observed in the case of the 1s degenerate mode, with a lower frequency shift between the two mechanical modes. Due to the difficulty to clearly differentiate the two peaks, and especially to control the removal of degeneracy, no such response is displayed in this paper. The thermomechanical model (see Eq. 5) seems well adapted in the case of the 1st degenerate mode, but is clearly not for the second response. A double peak model to therefore used to fit the PSD, without considering any mechanical mode coupling. For this specific example, it is effective as the two frequencies are sufficiently far from each other (lower mode coupling). Two dispersive optomechanical couplings are thus deduced, one for each mode, with values in the same order of magnitude than the previous measurements. However, a 10 times higher coupling is observed in the case of the (2, 1)/(1, 2) spectrum, which can be attributed to the degenerate nature of the mode. The basic function used to fit the spectrum does not consider the two mechanical contributions occuring at the same frequency. A more complex model should be used, to take into account the mechanical mode coupling between both, which is not negligible when the resonance frequencies are close enough to each other. In this situation, the extracted $g_{\text {om}}$ can be seen as an effective dispersive coupling: each mode is coupled to the intra-cavity optical field and contributes to the optomechanical interaction, which induces a higher coupling value.

### The periodic optomechanical extinctions

The MIM arrangement features a periodic behavior of the optomechanical couplings with the membrane position along the cavity axis^2^. When the mechanical resonator is disposed at a node of the stationnary intracavity field, the dispersive coupling vanishes. This intrinsic property is dynamically verified using the piezo material to precisely move the membrane across several nodes.

**Figure 5:**
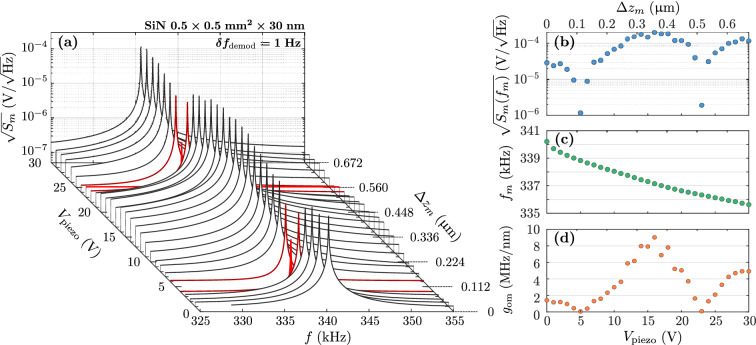
Observation of the periodic optical PSD extinctions on a SiN membrane ($0.5\times {0.5}\,{{\mathrm{mm}}^2}\times {30}\,{\mathrm{nm}}$): (**a** )Influence of the voltage ($V_{\text {piezo}}$) applied to the piezo material (or the membrane relative position $\Delta z_m$) on the fundamental optical PSD. Two extinctions are highlighted in red. Only the thermomechanical fit curves are displayed. (**b**)–(**d**) Respectively the optical PSD $\sqrt{S_m}$ at resonance, the resonance frequency, and the dispersive optomechanical coupling as a function of $V_{\text {piezo}}$ or $\Delta z_m$.

The fundamental thermomechanical spectrum is measured for each membrane position and the mechanical parameters extracted, for all the optomechanical cavities. The PDH stabilization can be maintained between each acquisition if the optical frequency shift induced by the piezo deformation is small enough (*i.e.* if the applied voltage step is sufficiently low). As an illustration, the results for the SiN membrane ($0.5\times {0.5}\,{{\mathrm{mm}}^2}\times {30}\,{\mathrm{nm}}$) are displayed in Fig. 5. The voltage applied to the piezo material is converted to a membrane relative displacement using the piezo sensitivity (${22.4}\,{\mathrm{nm/V}}$). In this example, the membrane is moved by almost $\lambda /2$. Two extinctions of the optical PSD, spaced of ${0.4}\,{\upmu \text {m}}$ ($\lambda /4$), are observed (see Fig. 5b). The amplitude at resonance is periodically reduced by two orders of magnitude, which reflects the position of a node and an antinode of the stationnary field. It dynamically indicates the vanishing of the dispersive coupling predicted by the static measurements (local maxima of the optical resonance frequency shift, see Fig. 1f). One can also observe a linear decrease of the mechanical resonance frequency, potentially due to two different effects. The most probable source is a local variation of the thermal environment of the membrane due to Joule heating of the piezo when applying a high voltage. The second possibility is a variation of the membrane constraints induced by the piezo deformation. Besides, this is coherent with the shift of the frequency gap induced by the piezo deformation observed on a higher order degenerate mode. Both hypothesis could explain the observed linear dependency of the resonance frequency on the applied voltage. Note that the corresponding quality factor does not exhibit any particular trend with the membrane position ($Q_m\approx 5\times 10^4$). The dispersive coupling is finally retrieved as a function of the membrane relative position (see Fig. 5c). The behavior of the dispersive coupling with $\Delta z_m$ is mirrored on the evolution of the amplitude at resonance with the two predicted extinctions. One could also confirm the MATE intrinsic property, observed with the basic optical characterizations, through the asymmetry of the coupling.

### Stability of the optomechanical signal

The stability of the optomechanical signal is finally investigated, for a low-stress SiN and a high-stress $\hbox {Si}_3\hbox {N}_4$ membranes. The PDH closed loop allows to maintain the laser emission frequency at the optical resonance $f_{\text {cav}}$ over several days. 200 acquisitions of the thermomechanical spectrum are done in a row, while always regularly checking if the laser is still maintained at optical resonance. To do so, the DC and the error signals are recorded during one minute, between each measurement. A given acquisition lasts approximately ${24}\,{\mathrm{min}}$. The whole characterization lasts around ${83.3}\,{\mathrm{h}}$, for each membrane. These measurements demonstrate an excellent stability without frequency noise degradation (low frequency noise maintained in the ${\mathrm{kHz}/\sqrt{{\mathrm{Hz}}}}$ range).

**Figure 6:**
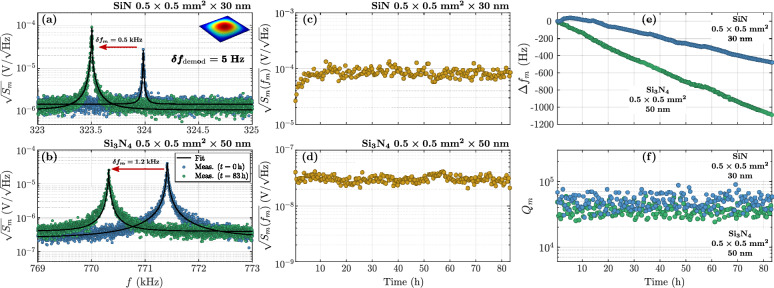
Long-term stability of the optomechanical signal. Two acquisitions of the optical PSD in ${\hbox {V}/\sqrt{\mathrm{Hz}}}$ of: (**a**) a SiN membrane ($0.5\times {0.5}\,{{\mathrm{mm}}^2}\times {30}\,{\mathrm{nm}}$), and (**b**) a $\hbox {Si}_3\hbox {N}_4$ membrane ($0.5\times {0.5}\,{{\mathrm{mm}}^2}\times {50}\,{\mathrm{nm}}$), nearby the fundamental resonance frequency, at different times of the same PDH stabilization closed loop. The black curves are the thermomechanical fits. (**c**, **d**) Extracted optomechanical signal at mechanical resonance for, respectively, the SiN and the $\hbox {Si}_3\hbox {N}_4$ membranes, as a function of the time. (**e**) Extracted resonance frequency shift $\Delta f_m$ and (**f**) mechanical quality factor $Q_m$ for both mebranes, as a function of the time.

The first and last optical PSD acquisitions are compared each other in Fig. 6a for the low stress SiN and (b) for the high stress $\hbox {Si}_3\hbox {N}_4$. The optical PSD at resonance is shown as a function of the time in Fig. 6c, d. A linear drift of the mechanical resonance frequency with time is observed (see Fig. 6e). This trend is most probably due to the tensile stress variations during the experiments. The frequency shifts $\delta f = {530}\,{\mathrm{Hz}}$ and ${1200}\,{\mathrm{Hz}}$ are observed after $83\,$h for, respectively, the SiN and the $\hbox {Si}_3\hbox {N}_4$ membranes. In other words, the slope of the frequency relative variations are close to $\delta f/f_0 = 2.05\times 10^{-5}\,{\hbox {h}^{-1}}$ for the SiN and $1.85\times 10^{-5}\,{\hbox {h}^{-1}}$ for the $\hbox {Si}_3\hbox {N}_4$. The corresponding relative in-plane tension variations are proportional to these relative frequency variations ($\delta {\mathcal {T}}/{\mathcal {T}} \propto 2\delta f/f_{11}$). They are therefore twice as big. At first glimpse, these stress variations might originate from thermal heating of membranes or surrounding pressure variation. However, the thermal time constant of the membrane with its silicon frame is rather close to $4\times 10^{-4}\,{\mathrm{s}}$ since the variation takes hours without any mechanical stabilization observed. In addition, the pressure variation cannot result in a frequency drift with such a slope. A quick calculation of the internal stress with the pressure leads to $10^{-3}\,{\mathrm{mbar}}$ of variation over $83\,$h for the SiN membrane for instance. Such a variation has not been observed when measuring the quality factor (see Fig. 6f). Moreover, as the pressure relative variation is proportional to the frequency relative variation it would mean a nominal pressure around ${30}\,{\mathrm{mbar}}$, which is also inconsistent with the pressure levels set between $10^{-6}$ and $10^{-5}\,{\mathrm{mbar}}$.

The stress and its behavior with time depend on several parameters that are the thickness, and deposition conditions (temperature, gas ratio, deposition rate, annealing). Adsorbed contaminants at the membrane surface can also induce a superimposed surface stress. A desorption mechanism occurs when the membranes are placed under vacuum which may explain the stress variation and the frequency changes.

## Discussion

A complete study of the optical, optomechanical and thermal features of this novel external lensed FBG-based MIM setup has been reported. With basic optical characterizations, high quality factor optical resonance are demonstrated, and the periodic behavior of the resonance conditon and decay rate is optically confirmed, indicating potential dispersive and dissipative interactions. The PDH frequency stabilization technique has been implemented, with an optimization procedure for low-frequency noise reduction. A rigourous, reliable and fast empirical method for the mechanical mode identification has been described, using the stabilized systems and the dispersive coupling. The thermomechanical spectrum of silicon nitride membranes, with various geometries and intrinsic constraints, have been studied. The MIM/MATE intrinsic behaviors have been dynamically confirmed. The excellent optical stability of the setup over time has been demonstrated by performing continuous measurements of the optomechanical spectrum over several days. Finally, our MIM architecture coupled to an efficient PDH stabilization method enables to offer a highly stable platform for sensing purpose, which would require long acquisition time and long-term stability. The laser and optical cavity remain locked for hours, allowing us to measure the mechanical spectrum automatically without manual setup adjustments.

In the prospect of optomechanical sensing^42^, various concrete applications could benefit from this MIM architecture. Among them, inertial^43^ (accelerometer/gyrometer), high resolution displacement and ultrasound^44^ sensing, with an optimized mechanical resonator design and geometry, seem to be relevant applications. However, our configuration is particularly promising for two other reasons.

Firstly, a neutral mass spectrometer with a nano resonator used as a gravimetric sensor has been demonstrated^45^. Our system could be used likewise for measuring several modal resonances of the membrane. The mechanical resonance frequency stability $\sigma _f$ and the limit of detection (LOD) are roughly estimated from the SNR with the expressions: $\sigma _f=(1/2Q_m)\times (1/SNR)\times \sqrt{1/\tau }$ where $\tau$ is the integration time, and $\text {LOD} = 2m_{\text {eff}}\times \sigma _f$, where $m_{\text {eff}}$ is the effective mass^46,47^. SiN membranes (low stress, ${30}\,{\mathrm{nm}}$ thick) exhibit a typical frequency stability close to $10^{-5}$ leading to a LOD close to ${0.12}\,{\hbox {pg}/\sqrt{\mathrm{Hz}}}$. The frequency stability is extracted from the thermomechanical noise, which is the predominant noise floor. It is necessary to decrease the size of both the cavity and the suspended membrane to improve the LOD. This setup would be efficient with ultra-thin membranes that could be made in Si or SiN below ${10}\,{\mathrm{nm}}$ or 2D-materials like graphene. Resonators with large suspended graphene membranes^48^ have recently been demonstrated. Such a system can be a good tradeoff between low LOD and a large capture section of molecules to analyze. Array of suspended membranes with spatial multiplexing could be included into the system to increase the analysis throughout.

Secondly, optomechanical setups have also been used for thermal sensing^49,50^. In the same way, the MIM structure is also a good alternative for building a resonant bolometer. Based on the frequency stability expected with our device, the minimum detectable stress is close to ${18}\,{\mathrm{kPa}}$. If we consider a differential relative dilatation (SiN versus Si) of $10^{-6}\,{\hbox {K}^{-1}}$^51,52^ the smallest detectable temperature variation would be close to ${100}\,{\mathrm{mK}}$. This rough estimation gives a first insight on the possible performance. To develop a real bolometer matrix, we have to adapt the MIM setup to work in the mid-infrared (mid-IR, $3-{15}\,{\upmu \text {m}}$) range. The membrane should have a good mid-IR absorption and the mechanical anchoring should have sufficient thermal insulation to insure reasonable thermal time constant (typically ${60}\,{\upmu \hbox {s}}$ per pixel).

## Methods

### Cavity construction and alignment

The lensed FBG-based cavity is a MIM setup based on a hybrid external fiber Fabry–Perot interferometer (see Fig. 1b). We used a commercial highly reflective lensed FBG (physical length around ${10}\,{\mathrm{mm}}$) from Raysung Photonics, as an input mirror, in front of a broadband dielectric mirror to build a basic Fabry–Perot. The uniform FBG is designed to exhibit a reflectivity above ${94}\,{\%}\pm {5}\,{\%}$ (measured values for multiple lensed FBGs from the same manufacturing process). The back mirror is a $1\,$inch fused silica broadband dielectric mirror (respectively, BB05-E04 or BB1-E04, Thorlabs), with a reflectivity above ${99}\,{\%}$ between 1280 and ${1600}\,{\mathrm{nm}}$. The lens is a conventional quarter-pitch GRIN lens designed to generate a beam waist of ${0.2}\,{\mathrm{mm}}$ at a working distance between 0.5 and ${1}\,{\mathrm{mm}}$. It is characterized by an anti-reflection coating on both side to prevent from any unwanted interferences induced by multiple reflections at the air-glass interfaces. The distance between the end of the FBG and the end-facet of the lens is ${7.6}\,{\mathrm{mm}}$, which imposes a lower limit for the achievable cavity length. We used a UV curing adhesive (Polytec UV 2195) to fixed the suspended silicon nitride membrane on a low voltage ring or square piezoelectric chip (respectively PA44LEW or PA4GKH5W, Thorlabs). The piezoelectric chip is then UV glued directly on the dielectric mirror, in order to passively align the membrane relatively to the back mirror. The main usage of the piezoelectric components is to precisely move the membrane along the cavity axis. The alignment setup was especially developped for vacuum measurements (see Fig. 1a): the lensed FBG is maintained on a steel GRIN lens holder (561-GR, Newport) on a short rail, itself fixed on a high resolution open loop 3-axis piezo-positionner (superposition of 3 linear stages Q-522.230, PI). Each stage is characterized by a ${2.6}\,{\mathrm{mm}}$ travel range and a ${4}\,{\mathrm{nm}}$ resolution. They are also suitable for low pressure working condition. The membrane-on-mirror assembly is placed on a stainless steel lens tube (SM1L05V for 1 inch mirror only, Thorlabs) which is fixed in a kinematic mount with 3 angle adjusters with ${5}\,{\upmu \hbox {rad}}$ resolution (POLARIS-K05T6 for 0.5 inch mirrors or POLARIS-K1T for 1 inch mirrors in lens tubes, Thorlabs). This positioner is attached using a $1\,$inch diameter cylindrical post, in front of the piezo-controlled 3-axis positioner. This approach ensures a good stability and displacement resolution. The dielectric mirror is characterized by a large optical bandwidth (${320}\,{\mathrm{nm}}$) compared to the FBG bandwidth (${0.5}\,{\mathrm{nm}}$). This served as a reference for the alignment of the membrane-on-mirror assembly and the lensed FBG: the main objective was indeed to maximise the signal reflected by the broadband mirror and coupled back into the fiber, outside of the FBG bandwitdh. The alignment was performed with a laser close to one of the FBG bandwidth edges, in order to increase the signal outside of the band relatively to the signal directly reflected by the fiber grating. The whole system was placed in a stainless steel cylindrical vacuum chamber designed to attain a pressure of $10^{-6}-10^{-5}\,{\mathrm{mbar}}$ with a primary pump coupled a thermomolecular pump (referenced TMH 071 P, Pfeiffer). The pumps are controlled with a dedicated electrical unit connected to a pressure gauge. The chamber has two vacuum compatible fiber coupled feedthroughs to send and extract the light.

### Implementation of the PDH closed loop

Our laser source is a commercial grating stabilized and tunable external-cavity diode laser from Toptica (DL pro), controlled by a dedicated unit (DLC-pro). It is a highly coherent laser diode emitting at ${1.55}\,{\upmu \text {m}}$ (linewidth below ${100}\,{\mathrm{kHz}}$). The emission wavelength can be tuned by a coarse adjuster over typically ${100}\,{\mathrm{nm}}$. The laser also includes a piezo actuator that controls the angle orientation of a diffraction grating within the laser cavity, to continuously and finely adjust the emission frequency. This is done by a applying a continuous voltage from 0 to ${140}\,{\mathrm{V}}$ with a sensitivity around ${0.2045}\,{\mathrm{GHz}/{\mathrm{V}}}$. It corresponds to a fine tunability window of ${28.63}\,{\mathrm{GHz}}$ in frequency or ${0.23}\,{\mathrm{nm}}$ in wavelength. The laser head includes an optical isolator (${65}\,{\mathrm{dB}}$ extinction) and a fiber coupling unit (Fiberdock, Toptica) mounted and pre-aligned by the manufacturer. The optical bench used to implement the PDH closed loop is sketched in Fig. 1c. An EOM is needed to generate the desired phase modulated input beam. We used a low frequency lithium niobate EOM (MPZ-LN, iXblue) which allows us to modulate the laser at frequencies below ${150}\,{\mathrm{MHz}}$. This EOM is supplied by an oscillating signal from a high frequency LIA (referenced UHFLI, Zurich Instruments) at the required frequency. In order to reach the optimal modulation depth, we used a RF amplifier (DR-VE-0.1-MO, iXblue). It enables a ${26}\,{\mathrm{dB}}$ amplification of signals oscillating up to ${200}\,{\mathrm{MHz}}$. A controller (FPC562, Thorlabs) was added between the laser source and the EOM, to ensure the light polarization corresponds to the one of the EOM. Directly after the modulator follows another passive element: the polarization maintaining optical circulator (CIR1550PM-APC, Thorlabs), to extract the light in reflection. Note that the working polarization is the same as for the EOM (slow axis pass and fast axis blocked). Another polarization controller was added before the cavity to maximize the reflected signal in the FBG bandwidth (also designed for slow axis operation). The reflected signal, redirected by the circulator, is measured by a low noise high bandwidth (up to ${200}\,{\mathrm{MHz}}$) photoreceiver (OE-300-IN-01, Femto). The oscillator, the phase shifter, low-pass filter and PID controller are included in the high frequency LIA, and are used to generate and isolate the PDH error through the *y* or *x* component of the demodulated signal. The correction is fed into the laser controller. The Analog Remote Control (ARC) is a multiplication factor that adjusts its amplitude and converts it into an offset. The resulting external signal is added to the initial laser piezo voltage (that control the diffraction grating within the laser) to correct the emission wavelength. The closed loop procedure involves four main steps. Firstly, a continuous forward and backward laser wavelength scan around the resonance peak is performed, with the phase modulation activated. The phase shift between the oscillator and the measured reflected signal is adjusted to ensure a zero shift at optical resonance and maximise the error signal amplitude on one of the demodulation component. Then, the error signal is fitted in the linear part to extract the slope. The continuous scan is then set off and the wavelength is manually moved to the optical resonance, until the DC response become lower than a given threshold. Finally the correction signal is fed into the laser to correct the laser frequency according to the error. This procedure has been automated using a homemade Python script to communicate with all the intruments.

### Thermomechanical calibration

We fitted every measured optical PSD using a thermomechanical model, following the method described in the paper of B.D. Hauer *et al.*^39^. This fit is important as it gives a physical meaning of all the characterization performed in this work. The random thermal motion of the mechanical resonator is derived using the fluctuation-dissipation theorem which results from the equipartition theorem. The external force in this case is generated by random fluctuations of the thermal environment (Langevin thermal force), which is well described by a white noise. The thermomechanical noise $S_{\text {th}}$ is deduced by considering a harmonic response of the mechanical resonator:

4 $$\begin{aligned} S_{\text {th}}(\omega ) = \dfrac{4k_BT}{m_{\text {eff}}} \dfrac{\omega _m/Q_m}{(\omega _m^2-\omega ^2)^2+\omega ^2 (\omega _m/Q_m)^2} \end{aligned}$$

where $k_B$ is the Boltzmann constant, *T* the temperature, $m_{\text {eff}}$ the effective mass and $\omega _m=2\pi f_m$ the mechanical resonance angular frequency. In practice, we had to take into account the other sources of noise, which we assumed to be also white. The fitting model $S_{\text {fit}}$ of our experimental optical PSD in voltage unit then reads

5 $$\begin{aligned} S_{\text {fit}}(\omega ) = S_N + \alpha \dfrac{\omega _m/Q_m}{(\omega _m^2-\omega ^2)^2+\omega ^2 (\omega _m/Q_m)^2} \end{aligned}$$

where $S_N$ is the constant noise PSD, $\alpha$ is a conversion factor, $\omega _m$ is the angular resonance frequency and $Q_m$ the mechanical quality factor. This model was applied on each measurement with $\omega _m$, $Q_m$, $\alpha$ and $S_N$ as fit parameters. This $\alpha$ factor is the most important parameter of the model as it allows a conversion of the measured PSD from voltage to mechanical displacement units, and therefore a calibration of the mechanical signal. It is relatively easy in a PDH configuration, where the detuning between the laser and the cavity remains null. We are going to detail this last remark with several equations in order to confirm the interest of the factor $\alpha$, and relate it to the coupling strength $g_{\text {om}}$. We thus consider a voltage thermal fluctuations measured by our photodetector and denoted by $\delta V_{\text {V,\,th}}$. It is related to the cavity frequency fluctuations $\delta f_{\text {cav}}$ through

6 $$\begin{aligned} \delta V_{\text {V,\,th}}= & {} s_{\text {err}}\delta f_{\text {cav}},\nonumber \\= & {} s_{\text {err}}g_{\text {om}}\delta z_m, \end{aligned}$$

where $s_{\text {err}}$ is the error signal sensitivity in ${\mathrm{V/Hz}}$ and $\delta z_m$ is the mechanical displacement. The measured optical PSD in voltage units then reads

7 $$\begin{aligned} S_{\text {V,\,th}} = s_{\text {err}}^2g_{\text {om}}^2 S_m, \end{aligned}$$

where $S_m$ is the mechanical PSD in ${{\mathrm{m}}^2/{\mathrm{Hz}}}$. Using the previous relationship and Eqs. (4) and (5), we confirm the fit parameter $\alpha$ can indeed be understood as a conversion factor of the PSD from units of ${\hbox {V}^2/{\mathrm{Hz}}}$ to ${\hbox {m}^2/\mathrm{Hz}}$:

8 $$\begin{aligned} \mathop {\mathop {\alpha /s_0}_{}}_{ {[\hbox {V}^2/{\mathrm{m}}^2]} } = s_{\text {eff}}^2g_{\text {om}}^2 \end{aligned}$$

where $s_0 = 4k_BT/m_{\text {eff}}$. Therefore the displacement spectrum is given by

9 $$\begin{aligned} \mathop {\mathop {\sqrt{S_m}}_{}}_{ {[{\mathrm{m}}/\sqrt{{\mathrm{Hz}}}]} } = \dfrac{1}{s_{\text {err}}g_{\text {om}}} \sqrt{S_V} = \dfrac{1}{\alpha /s_0} \mathop {\mathop {\sqrt{S_V}}_{}}_{ {[\hbox {V}/\sqrt{\mathrm{Hz}}]} } \end{aligned}$$

Using this thermomechanical fit, the dispersive optomechanical coupling value can therefore be quantified. We used a “trust-region algorithm” to fit our measurements. The precision of the estimation of the mechanical parameters is $\pm 0.02$ to ${0.2}\,{\mathrm{Hz}}$ for the frequency, $\pm {10}\,{\%}$ of $Q_m$ for the quality factor, and in the order of $\pm {2}\,{\%}$ of $g_{\text {om}}$ for the dispersive coupling. Note that the calibration does not required any dissipative coupling, as the error signal indirectly measures the optical phase changes *i.e.* the shifts of the optical resonance frequency.

## Data Availability

The data that support the plots within this paper and other findings of this study are available from the corresponding authors upon reasonable request.
